# Enhancement of cAMP-mediated inotropic responses by CNP is regulated differently by PDE2 in normal and failing hearts

**DOI:** 10.1186/2050-6511-16-S1-A66

**Published:** 2015-09-02

**Authors:** Silja Meier, Kjetil Wessel Andressen, Jan Magnus Aronsen, Ivar Sjaastad, Tor Skomedal, Jan-Bjørn Osnes, Eirik Qvigstad, Finn Olav Levy, Lise Román Moltzau

**Affiliations:** 1 Department of Pharmacology, University of Oslo and Oslo University Hospital, Oslo, Norway; 2 K.G. Jebsen Cardiac Research Centre and Center for Heart Failure Research, University of Oslo, Oslo, Norway; 3 Institute for Experimental Medical Research, University of Oslo and Oslo University Hospital, Oslo, Norway; 4 Bjørknes College, Oslo, Norway

## Background

Natriuretic peptide levels are increased in heart failure (HF). Atrial (ANP) and brain (BNP) natriuretic peptide mediate their effects preferentially through the natriuretic peptide receptor (NPR)-A, and C-type natriuretic peptide (CNP) through NPR-B. NPRs are membrane bound guanylyl cyclases that increase cyclic GMP (cGMP) production when activated. We have previously shown that NPR-B stimulation by CNP enhances β_1_-adrenoceptor (β_1_-AR)- and 5-HT_4_ serotonin receptor-mediated signaling in failing hearts, probably through inhibition of phosphodiesterase (PDE) 3, a potential detrimental effect in the failing heart. In this study we examine the role of PDE2 in regulating the CNP-induced enhancement of β_1_-AR and 5-HT_4_ signaling in non-failing (Sham) and failing (HF) hearts, as PDE2 is a dual-selective PDE and can potentially be stimulated by cGMP.

## Methods

Chronic heart failure was induced in male Wistar rats by 6-week coronary artery ligation. Contractility studies were performed *ex vivo* in left ventricular muscle strips in the presence of appropriate receptor agonist and antagonists. cGMP measurements were performed on isolated left ventricular cardiomyocytes and PDE activity assays on left ventricular cardiomyocyte homogenates.

## Results

CNP, by stimulating NPR-B, was able to enhance β_1_-AR- and 5-HT_4_-mediated inotropic responses in Sham and HF left ventricular strips. CNP elicited a similar increase of cGMP in cardiomyocytes from Sham and HF. cGMP reduced the cAMP-PDE activity of PDE3 and increased the cAMP-PDE activity of PDE2 concentration-dependently in cardiomyocyte homogenates in a similar way in Sham and HF. In Sham inhibition of PDE2 by EHNA amplified the CNP-induced enhancement of β_1_-AR- and 5-HT_4_-mediated inotropic responses. In HF PDE2 inhibition did not influence the functional effects of CNP despite increasing the cGMP response to the same marked extent as in Sham.

## Conclusions

There is a preserved mechanism of CNP-induced enhancement of β_1_-AR and 5-HT_4_ in Sham and HF. cGMP levels and cGMP-mediated activation and inhibition of cAMP-PDE activity of PDE2 and PDE3, respectively, are similar in Sham and HF. However, PDE2 seems more involved in regulating the β_1_-AR and 5-HT_4_ enhancement in Sham compared to HF, which might reflect differences between Sham and HF in PDE2 expression or compartmentation.

